# The Influence of State and Trait Anxiety on the Achievement of a Virtual Reality Continuous Performance Test in Children and Adolescents with ADHD Symptoms

**DOI:** 10.3390/jcm10122534

**Published:** 2021-06-08

**Authors:** Débora Areces, Celestino Rodríguez, Trinidad García, Marisol Cueli, Paloma González-Castro

**Affiliations:** Faculty of Psychology, University of Oviedo, Plaza Feijoo s/n, 33003 Oviedo, Spain; arecesdebora@uniovi.es (D.A.); garciatrinidad@uniovi.es (T.G.); cuelimarisol@uniovi.es (M.C.); mgcastro@uniovi.es (P.G.-C.)

**Keywords:** ADHD, virtual reality, continuous performance test, anxiety, STAI-C scale, ADHD comorbidity

## Abstract

The three types of presentations of ADHD often co-occur with other disorders, anxiety being one of the most prevalent. For this reason and because there are few studies that have examined the influence of anxiety on attentional activities, this study aims to determine how internalizing difficulties (anxiety levels) can influence performance in a virtual reality continuous performance test. The study used a non-probabilistic clinical sample comprising 68 boys (66%) and 35 girls (34%) aged between 6 and 16 (*M* = 12.24; *SD* = 2.45) who had been referred to clinical services for the evaluation of ADHD symptoms. Once informed consent was given, the children were administered the STAI-C scale and a virtual reality continuous performance test by expert researchers. Hierarchical regression models showed that only state anxiety demonstrated significant explanatory power over attentional variables. These findings confirm how important it is for children to feel relaxed when they undergo psychological evaluation tests, as otherwise the individual’s intervention design would be based on biased data. Similarly, the findings also suggested an effect of IQ in the interpretation of continuous performance scores.

## 1. Introduction

Attention deficit and hyperactive disorder (ADHD) is a common neurobehavioral disorder in childhood with high prevalence of the symptoms persisting into adulthood [1,2,3]. The disorder is characterized by a series of more or less stable symptoms, such as hyperactivity, impulsivity, and attention deficit, which usually have negative consequences in family relationships, academic performance, and social and work-related functioning [4]. Moreover, ADHD has been shown to often co-occur with other disorders. According to international studies [5,6], half of the children with ADHD have oppositional defiant disorder, 33% have an anxiety disorder, and another 33% have dysthymic disorder or major depression. Along similar lines, studies have also demonstrated that children with ADHD are more likely to exhibit depression and/or anxiety than their peers [7]. Other studies have shown that these children exhibit dysregulation of negative emotions such as sadness or frustration [8]. Such high rates of comorbidity make it very difficult to perform a differential diagnosis or to carry out an intervention suited to each child’s particular situation. The comorbidity of ADHD with other mental disorders (such as anxiety or depression) has been associated with significant disability and poorer quality of life, as well as impaired social and family functioning [9,10].

In terms of ADHD presentation, it is worth noting that ADHD presentations demonstrate notable comorbidity with internalizing disorders, with anxiety disorder possibly more common in the predominantly inattentive ADHD presentation than in the combined ADHD presentation [11]. Internalizing disorders consist of mental disorders whose primary symptoms involve inner emotions as opposed to outward behavior [12]. Internalizing problems can manifest in a variety of ways, such as anxiety, depression, low self-esteem, somatic complaints without a known medical basis, and social withdrawal from contact [13].

Currently, there are few empirical studies which have attempted to explore the causes of this comorbidity of ADHD and anxiety. More specifically, with regard to the etiology of the comorbidity of ADHD and anxiety, current research indicates that ADHD and anxiety disorders seem to have independent genetic factors, and there is no common genetic component [5,6]. Along similar lines, other studies indicate that people diagnosed with ADHD and anxiety disorder exhibit a completely different profile than people who present these disorders in isolation [8,9,10]. The most widely accepted hypothesis in all of the studies about the co-occurrence of ADHD and anxiety is that anxiety may lead to intrusive emotions or thoughts—internal distractors—that significantly affect the patient, such as inattention or hyperactivity/impulsivity symptoms [14]. One important aspect to consider in clinical practice is that treating one disorder can interfere with the other. The US Food and Drug Administration warns that stimulant medications may produce increased anxiety [15]. In fact, some studies have found that higher levels of anxiety are associated with a poorer pharmacological response (specifically, a poorer response to methylphenidate) [16,17]. Schatz and Rostain [18] showed that anxiety in ADHD may partially inhibit impulsivity and make working memory deficits worse. A recent study [16] noted that high levels of anxiety can significantly change the type of presentation, the progression of the symptoms, and the treatment of ADHD. More particularly, the presence of anxiety disorders could reduce the typical inhibitory dysfunction during childhood, increase the deficit in working memory in adolescence, and lead to sleep problems in adulthood [19]. A study by González-Castro et al. [20] analyzed the influence of trait and state anxiety depending on ADHD presentation and concluded that the combined presentation demonstrated higher trait anxiety, whereas the inattentive presentation demonstrated higher state anxiety. However, although some studies have focused on examining the influence of anxiety levels on ADHD symptoms and pharmacological response, none of them have analyzed the influence of anxiety by a continuous performance test. In this regard, the innovation of the present study is in pursuing a thorough understanding of how levels of anxiety (state or trait) can explain the results in a virtual reality-based continuous performance test. 

Achieving this objective would help clinicians understand how anxiety (state vs. trait anxiety) affects activities in which attentional capacity and inhibitory control play a role. In addition, thanks to the use of virtual reality, the results from the present study will have greater ecological validity than traditional tools because the test replicates a typical classroom with external (visual and auditory) distractors that are common at school (whispers, external noises, classmates talking, etc.) [21,22]. A recent study [21] showed that a virtual reality continuous performance test (called Aula Nesplora) not only allowed differentiation between a control group and ADHD groups, but also differentiated between the three types of presentations of ADHD (inattentive, impulsive–hyperactive, and combined presentation). Moreover, comparing diagnostic efficacy between a virtual reality continuous performance test (Aula Nesplora) and traditional continuous performance tests (tests based on the Go/NoGo paradigm without virtual reality), results have shown that the innovative virtual reality test is more effective in assessing ADHD symptoms and the results have greater ecological validity (since the patients’ behavior is very similar to their behavior in daily life) [23]. In this regard, the closest precedent of the Aula Nesplora test would be the so-called “Virtual Reality Classroom” [24]. In fact, Aula Nesplora follows the same logic as its predecessor, in the sense that it presents a task of sustained attention and inhibition of responses in the context of a virtual classroom. Moreover, this tool is adapted to the Spanish language and provides some interesting measures different from traditional continuous performance tests like the variable “attention focus quality,” which allows us to know the quality of the child’s attention when he or she is focused on the visual stimuli (in other words, the quality of attention when the child is not distracted and looks at the virtual whiteboard) [21]. These findings highlight how virtual reality continuous performance tests bring many benefits to the assessment of ADHD symptoms compared to traditional versions [21,22,23,24,25,26].

Moreover, research has also shown that VR rehabilitation interventions demonstrate significant improvements in attention, impulsive, and hyperactivity symptoms [25,26]. 

## 2. Materials and Methods

### 2.1. Participants

**Figure 1:**
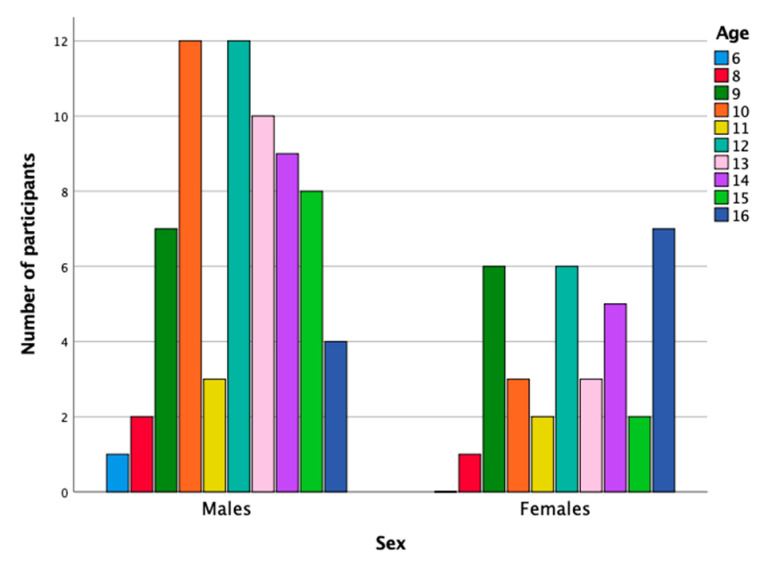
Sample distribution.

This study used a non-probabilistic clinical sample comprising 68 boys (66%) and 35 girls (34%) aged between 6 and 16 years (*M* = 12.24; *SD* = 2.45) with a mean IQ of 106.36 (*SD* = 14.38), who had been referred to clinical services for assessment of ADHD symptoms. The IQ was measured using the WISC IV scale [23]; subjects scoring below 80 or above 130 were removed from the sample. Figure 1 shows the sample distribution based on age and sex.

#### Inclusion Criteria

To be included in the study, the subjects had to meet the following requirements based on DSM-5 criteria [4]: (1) there must be a persistent pattern of inattention and/or hyperactivity–impulsivity that has lasted at least six months; (2) the inattention and/or hyperactive–impulsive symptoms must interfere with functioning or developmental level; (3) these symptoms must have been presented before the subject was 12 years old; (4) several symptoms must be present in two or more settings (such as at home or school; with friends or relatives; in other activities); (5) there must be clear evidence that the symptoms interfere with, or reduce the quality of, social, school, or work functioning; (6) the symptoms must not be better explained by another mental disorder. Additionally, none of the participants must be under medical treatment.

This evaluation was carried out by a clinician to verify the inclusion of the participants in the study.

### 2.2. Instruments

The WISC-IV by Wechsler [27] is a tool that assesses individual intelligence in children and adolescents between the ages of 6 years and 16 years 11 months. This is an individually administered test composed of 15 subtests that provide information on specific cognitive areas: verbal comprehension, perceptual reasoning, working memory and processing speed. In the present study, only the total intelligence quotient (TIQ) was considered. 

**Figure 2:**
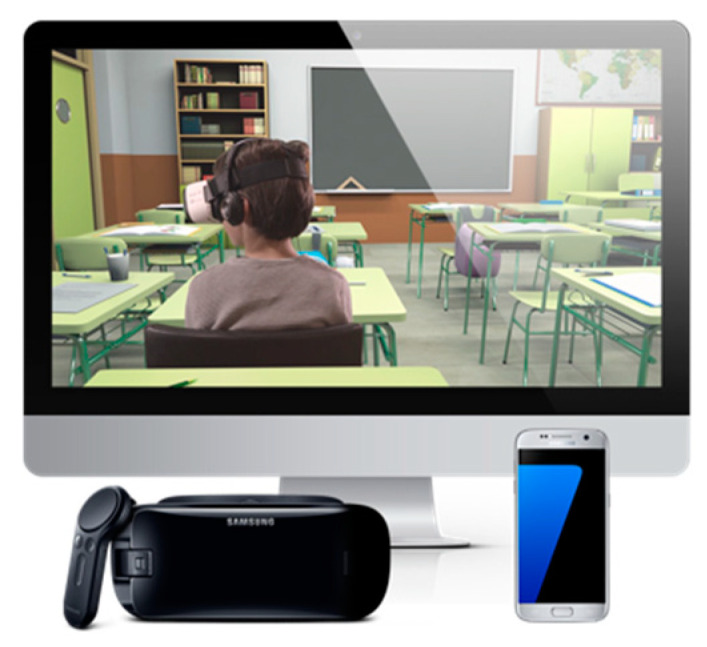
Aula Nesplora (virtual reality continuous performance test) hardware.

Aula Nesplora is a virtual reality-based continuous performance test [28], which has been shown to be reliable in the assessment of ADHD symptoms and has good ecological validity [21,22,23]. This tool measures attention, impulsivity, processing speed, and motor activity in participants between 6 and 16 years old. The task is performed in a virtual reality environment through three-dimensional (3D) glasses (head-mounted display (HMD)) equipped with a mobile phone (which is inserted within the 3D glasses) and headphones. The virtual stage presented through the HMD, following its predecessor [24], is similar to a classroom. The participant takes the perspective of a student sitting at one of the desks and facing the blackboard. The motor activity is based on head movements, which are detected by sensors in the mobile phone software. The software updates the angle of vision, giving the participant the feeling of actually being in a virtual school (Figure 2 shows the hardware necessary to perform the Aula Nesplora test).

The test consists of two main phases that are explained by a virtual teacher. The first task is based on the “x-no” paradigm (traditionally known as “no-go”) in which the participant must press a button as long as they do not see or hear the stimulus “apple.” There is also an “x” paradigm (or “go”) where the participants are asked to press a button whenever they see or hear the number “seven.” Once the test is complete, the AULA software provides a clinical report based on the following variables:

- Omissions: These are errors that occur when the participant should respond to the target stimulus but does not do so. It is a measure related to selective and focused attention.
- Commissions: These occur when the participant clicks on the button, even if the target stimulus has not been presented. This measure correlates with a lack of motor control or response inhibition.
- Response time: Average response time is the reaction time in milliseconds, used as a measure for processing speed.
- Motor activity: The 3D glasses used in this test have a motion sensor that records the full motor activity of the participant during the test. In this manner, head movements are captured to record frequency and relevance (i.e., required vs. unnecessary movements).

These variables are provided in general terms (the mean values from the total of the tasks performed) and differentiated by sensory channel (auditory or visual), contextual characteristics (presence and absence of distractors), and by the type of activity (go vs. no-go tasks). In this regard, the report offers useful data for creating modified interventions based exclusively on the patient’s own performance. The Cronbach alpha index for the present study gave excellent results (α = 0.928). In this study, only the general variables (the mean values of the following variables: omissions, commissions, response time, and motor activity) were considered because the objective was to examine how anxiety affects attention capacity in general terms (which means assessing the results for the tasks as a whole rather than only for a particular task).

The State-Trait Anxiety Inventory for Children (STAI-C) [29] consists of two 20-item scales that measure state and trait anxiety in children between 8 and 14 years old. The state anxiety scale examines anxiety as a temporary reaction to specific events or situations. It prompts the child to rate 20 statements on a 3-point scale ranging from “hardly ever true” to “often true.” The trait anxiety scale refers to the stable tendency to deal with experience and report negative emotions, such as fears, worries, and anxiety, across many situations. Higher scores in the STAI-C correspond to higher levels of anxiety. In the present study, a reliability index was calculated (Cronbach alpha), which gave good results (α = 0.728).

### 2.3. Procedure

Online meetings were held initially to obtain collaboration from clinical centers. Once the centers agreed to participate, researchers from the project were put in contact with the children’s families to answer any questions and resolve any issues about the assessment process. Once parental consent was given, both tests (STAI-C scale and the virtual reality continuous performance test) were conducted by expert researchers according to the study aims. The study was approved by the relevant Ethics Committee of the Principality of Asturias (reference: CPMP/ICH/135/95; code: TDAH-Oviedo), and all procedures were in compliance with relevant laws and institutional guidelines.

### 2.4. Data Analysis

The descriptive statistics for the study variables were analyzed (variables from the Aula Nesplora test and variables from the STAI-C scale), paying particular attention to skewness and kurtosis. Kline’s criterion was followed [30], according to which the maximum scores accepted for skewness and kurtosis range between 3 and 10. As the data obtained were within these limits, it was possible to carry out parametric analyses (using the percentile scores produced by Aula Nesplora and STAI-C). In order to control for the effects of age and sex in the variables analyzed, various ANOVAs were performed, with sex and age as independent variables, and the variables provided by STAI-C and Aula Nesplora as dependent variables. In addition, Pearson bivariate correlations were also calculated to analyze whether there was a relationship between anxiety variables (state and trait anxiety) and attentional and inhibitory control variables (omissions, commissions, response time and motor activity).

Following this, and once the existence of a significant relationship between attentional variables and anxiety levels was confirmed, four hierarchical regression analyses were performed to examine the predictive value of state and trait anxiety on the variables produced by Aula Nesplora (omissions, commissions, response time, and motor activity). Each hierarchical regression analysis included two models: model 1, composed of general variables (such as IQ, age, and sex), considering “sex” as a categorical variable (it was necessary to convert it into a dummy variable taking female as a reference); and model 2, which included the variables from model 1 and added the state and trait anxiety indexes from the STAI-C scale. The dependent variables for each hierarchical regression model were attentional variables: omissions, commissions, response time, and motor activity.

All statistical analyses were performed using SPSS v24.0 [31] with a significance level of *p* < 0.05.

## 3. Results

### 3.1. Preliminary Analysis

Table 1 shows the bivariate Pearson correlations between attentional variables provided by the VR continuous performance test and anxiety variables (state and trait anxiety).

As the results of the bivariate Pearson correlations in Table 1 show, the strongest correlations between attentional variables were found in omissions and response time. It is useful to note that both state and trait anxiety variables demonstrated significant bivariate correlation. Finally, on analyzing the correlations between attentional and anxiety variables, state anxiety demonstrated significant bivariate correlations with the scores in omissions, commissions, and response time. Trait anxiety only demonstrated significant bivariate correlation with scores for commission errors. 

Moreover, considering the high standard deviation with the STAI-C and motor activity variables, *M-estimators* were also calculated separately for boys (Huber’s *M-estimator* for state anxiety = 28.87; Huber’s *M-estimator* for trait anxiety = 52.63; and Huber’s *M-estimator* for motor activity = 49.04) and girls (Huber’s *M-estimator* for state anxiety = 14.85; Huber’s *M-estimator* for trait anxiety = 39.39; and Huber’s *M-estimator* for motor activity = 38.26).

Table 1 also shows that boys had higher scores in all the variables examined (attentional and anxiety scores). Higher scores in these variables indicate poorer performance. 

Table 2 shows the descriptive statistics (mean, standard deviation, asymmetry and kurtosis statistics) and the ANOVAs carried out to examine the differences depending on the sex and the age of the participants (considering the age variable as a quantitative and discrete variable).

As Table 2 shows, the highest scores in the attentional variables were for omission errors. On similar lines, looking at the results from the STAI-C scale (trait anxiety vs. state anxiety), the scores in the state anxiety scale were lower than those in the trait anxiety scale.

### 3.2. Regression Analysis

Following the analysis of the descriptive statistics, four hierarchical regression models were performed (Table 3) to determine the explanatory power of general variables (sex, age, and IQ) and anxiety variables (trait anxiety vs. state anxiety) over the attentional variables from the virtual reality (VR) continuous performance test (omissions, commissions, response time, and motor activity), which are related to ADHD symptomatology.

Table 3 shows that in model 1 (general variables such as sex, age, and IQ) only IQ demonstrated significant explanatory power for predicting omission errors. Gender and age demonstrated significant explanatory power for predicting commission errors (with a statistically significant explanatory power of 12%). Model 2 showed that state anxiety levels only demonstrated significant explanatory power to predict two of the total four attentional variables: omission (with a total explained variance of 13.7%) and commission errors (with a total explained variance of 27.3%). It is also worth noting that model 2 showed a significant increase in the explained variance, with the explained variance in the prediction of omissions increasing by 6.5% and the explained variance in the prediction of commissions increasing by 15.3%. Neither model 1 nor model 2 was significant in predicting response time and motor activity. Considering this, it is important to note that only state anxiety levels (rather than trait anxiety) from the STAI-C scale were shown to notably influence performance in a VR continuous performance test in which children must use attention and inhibitory control skills. 

## 4. Discussion

The present study aimed to determine the influence of anxiety levels on attentional variables (provided by a virtual reality continuous performance test). The Pearson bivariate correlations demonstrated significant correlation between state vs. trait anxiety and attentional variables and vice versa. These findings are in line with the study by González-Castro et al. [20], which indicated a significant correlation between anxiety levels and attentional variables provided by the D-2 test (a test which measures selective attention) and vice versa. On similar lines, the ANOVA results showed that boys had significantly worse results than girls in the commission errors variable. This may be due to the neurological differences between boys and girls in the early years [32]. The ANOVAs also showed that over the years the numbers of omission and motor hyperactivity errors improved significantly. This finding may be explained by the fact that attention depends on the level of automatization of various cognitive processes, and this automatization is positively related to age [33]. 

In addition, and in pursuit of the most important objective in the present study, the explanatory power of anxiety (more specifically, state and trait anxiety) was examined in terms of predicting performance in attentional variables from a VR continuous performance test. The hierarchical regression models showed that only state anxiety was able to significantly predict the values of two attentional variables: omission and commission errors. Considering previous studies [21,22] that reported that high numbers of omissions are related to inattention symptoms and that high numbers of commission errors are associated with impulsive behavior, this may suggest that in the sample in the present study, inattention and impulsivity were influenced by state anxiety levels. This emphasizes the importance of considering levels of anxiety during the assessment of ADHD symptoms, since sometimes a subject may be making omission or commission errors because of state anxiety levels rather than really having ADHD symptoms. These findings agree with previous studies that showed the influence of anxiety on ADHD symptomatology [19,20] and particularly studies showing that the presence of anxiety in patients with ADHD can affect the type of presentation. A psychological evaluation that detects comorbid problems has clear benefits not only for effective psychological therapy but also in considering the response to pharmacological treatment [14,15,16,33]. In this regard, one study by Jensen et al. [34] speculated that children who present ADHD and anxiety disorders might exhibit less impaired cognitive performance than those with ADHD alone. Similarly, Korenblum et al. [35] reported that children with ADHD and anxiety did not differ from those presenting only ADHD (without any comorbid disorders) in terms of response inhibition. However, unlike these studies, which considered anxiety in general terms (not differentiating between trait anxiety and state anxiety levels), the results of the present study are novel because they indicate that although state anxiety levels partially explained the results, the levels of trait anxiety did not demonstrate significant explanatory power in the prediction of any attentional variables from the VR continuous performance test. 

This study also showed that the patients’ general IQ demonstrated significant predictive power over performance in the omission variable. This result is in line with the study by Park et al. [36], which stated that mental health professionals must consider the effect of IQ on the interpretation of continuous performance Scores. The fact that attentional scores are within the normal curve does not always rule out a diagnosis of ADHD. Therefore, it is important to highlight that omission errors could be influenced by certain sub-items of IQ which are related, in turn, to sustained attention.

Finally, future research lines should address some of the limitations of the present study. Firstly, the sample size should be increased, and patients should be differentiated by their ADHD presentation (predominantly inattentive, predominantly impulsive, and the combined presentation). This would allow more specific conclusions to be made based on DSM-5 criteria. Similarly, the results should be compared to control groups with similar characteristics. This comparison would be useful in measuring the influence of anxiety according to ADHD diagnosis and considering the presence or absence of ADHD symptoms. In addition, another notable limitation suggests including more different types of variables such as (1) sociodemographic variables (e.g., the family’s socioeconomic status); (2) variables related to the mental health of the patients (use of medications, mood state and psychiatric comorbidities); and cognitive variables (working memory, processing speed), which can significantly affect ADHD symptoms, and therefore also the results from the virtual reality continuous performance test. For this reason, future studies should not only increase sample sizes but also add more information related to psycho-social variables. 

## 5. Conclusions

The findings of the present study have notable clinical implications, including the following: (1) increased age and IQ are related to better attentional performance; (2) in general terms, girls have better results than boys in attentional variables; (3) it is important to consider anxiety levels during the assessment of ADHD symptoms.

## Figures and Tables

**Table 1:** Bivariate Pearson correlations between attentional and anxiety variables.

Variables	1	2	3	4	5	6
Attentional						
1. Omissions	1	0.422 **	0.453 **	0.319 **	0.267 **	0.091
2. Commissions		1	0.355 **	0.336 **	0.433 **	0.233 *
3. Response Time			1	0.333 **	0.302 **	0.114
4. Motor Activity				1	0.183	0.057
Anxiety						
5. State Anxiety					1	0.304 **
6. Trait Anxiety						1
*M* Girls *(SD)*	54.62 (24.39)	46.71 (31.01)	48.79 (25.85)	40.56 (31.92)	23.64 (26.75)	42.38 (30.04)
*M Boys* *(SD)*	60.90 (24.60)	60.21 (26.42)	52.29 (31.22)	49.46 (34.86)	33.77 (30.04)	51.54 (32.37)

***Note***. * *p* < 0.05; ** *p* < 0.01 (2-tailed).

**Table 2:** Descriptive statistics and differences across age and sex for attentional variables and state/trait anxiety levels.

Variables	*M*	*SD*	Asymmetry	Kurtosis	Age Differences		Sex Differences	
					*F*(1102)	η_p_2	*F*(1102)	η_p_2
Attentional								
Omissions	58.80	24.59	−0.258	−0.992	1.741	0.161	1.485	0.050
Commissions	55.71	28.60	−0.260	−1.056	3.010 **	0.249	5.261 *	0.001
Response Time	51.13	29.45	0.066	−1.240	0.914	0.091	0.088	0.015
Motor Activity	46.49	34.01	0.142	−1.441	2.345 *	0.205	1.559	0.050
Anxiety								
State Anxiety	30.40	29.25	0.989	−0.299	1.518	0.143	2.767	0.027
Trait Anxiety	48.49	31.76	0.042	−1.262	0.687	0.070	1.902	0.019

***Note.*** ** *p* < 0.01; * *p* < 0.05.

**Table 3:** Hierarchical regression models for predicting attentional variables from VR continuous performance test.

Regression Models		Omissions	Commissions	Response Time	Motor Activity
1	Constant	119.191 (4.911 ***)	142.711 (5.219 ***)	92.673 (3.120 **)	82.090 (2.388 *)
	Sex (female)	−5.520 (−1.096)	−11.951 (−2.107 *)	−3.149 (−0.511)	−7.563 (−1.061)
	Age	−0.544 (−0.539)	−2.867 (−2.521 **)	−1.267 (−1.026)	−2.040 (−1.427)
	IQ	−0.437 (−2.574 **)	−0.338 (−1.767)	−0.205 (−0.987)	−0.011 (−0.045)
	*R* ^2^	0.072	0.120	0.021	0.035
	*F*(3, 102)	2.573	4.520 **	0.694	1.180
2	Constant	104.812 (4.325 ***)	117.886 (4.577 ***)	80.123 (2.653 **)	70.424 (2.001 *)
	Sex (female)	−3.144 (−0.630)	−7.611 (−1.436)	−1.007 (−0.167)	−5.729 (−0.797)
	Age	−0.161 (−0.161)	−2.268 (−2.139 *)	−0.950 (0.446)	−1.705 (−1.177))
	IQ	−0.451 (−2.704 **)	−0.377 (−2.128 *)	−0.222 (0.289)	−0.016 (−0.066)
	State Anxiety	0.207 (2.412 **)	0.334 (3.656 ***)	0.174 (0.107)	0.178 (1.425)
	Trait Anxiety	0.035 (0.447)	0.118 (1.424)	0.047 (0.631)	0.005 (0.046)
	*R* ^2^	0.137	0.273	0.056	0.057
	Δ*R*^2^	0.065	0.153	0.035	0.022
	*F*(5, 102)	3.089 **	10.144 ***	1.157	1.165

***Note.*** Values in the table are the β regression coefficient, and those in brackets are the Student *t*. *R*^2^ = variance explained; the variable “sex” is a dummy variable in which female sex has been taken as a reference; Δ*R*^2^ = change in variance explained. *** *p* < 0.001, ** *p* < 0.01, * *p* < 0.05
